# Endangered Przewalski’s Horse, *Equus przewalskii*, Cloned from Historically Cryopreserved Cells

**DOI:** 10.3390/ani15050613

**Published:** 2025-02-20

**Authors:** Ben J. Novak, Oliver A. Ryder, Marlys L. Houck, Kelcey Walker, Lexie Russell, Blake Russell, Shawn Walker, Sanaz Sadeghieh Arenivas, Lauren Aston, Gregg Veneklasen, Jamie A. Ivy, Klaus-Peter Koepfli, Anna Rusnak, Jaroslav Simek, Anna Zhuk, Andrea S. Putnam, Ryan Phelan

**Affiliations:** 1 Revive & Restore, 1505 Bridgeway #203, Sausalito, CA 94965, USA; ryan@reviverestore.org; 2 Beckman Center for Conservation Research, San Diego Zoo Wildlife Alliance, Escondido, CA 92027, USA; oryder@sdzwa.org (O.A.R.);; 3 ViaGen Pets & Equine, 715 Discovery Blvd #410, Cedar Park, TX 78613, USAlexie.russell@okstate.edu (L.R.);; 4 Timber Creek Veterinary Hospital, 19302 Farm to Market Rd 1541, Canyon, TX 79015, USA; govenny@aol.com; 5 Independent Researcher, Erie, CO 80516, USA; 6 Smithsonian-Mason School of Conservation, George Mason University, 1500 Remount Road, Front Royal, VA 22630, USA; koepflik@si.edu; 7 Center for Species Survival, Smithsonian’s National Zoo and Conservation Biology Institute, 1500 Remount Road, Front Royal, VA 22630, USA; 8 Institute of Applied Computer Science, ITMO University, 197101 St. Petersburg, Russiaania.zhuk@gmail.com (A.Z.); 9 Prague Zoo, 171 00 Prague, Czech Republic; 10 Laboratory of Amyloid Biology, St. Petersburg State University, 199034 St. Petersburg, Russia; 11 Dallas Zoo, 650 S R.L. Thornton Fwy, Dallas, TX 75203, USA

**Keywords:** somatic cell nuclear transfer, cloning, genetic rescue, Przewalski’s horse, biobanking

## Abstract

Cloning from historically cryopreserved cells offers a potential means to restore lost genetic variation or increase the representation of particular lineages within bottlenecked species, provided such biobanked materials are archived for such genetic rescue applications. One species for which cloning can provide genetic management benefits is Przewalski’s horse, *Equus przewalskii*. All ~1800 living Przewalski’s horses, distributed across ex situ breeding facilities and in situ reintroduction sites, are descended from one or more of the five founder lineages established by the 12 horses captured from the wild between 1898 and 1947. Since the 1970s, the San Diego Zoo Wildlife Alliance Biodiversity Bank’s Frozen Zoo^®^ (Escondido, CA, USA) has biobanked cells or tissues of 575 individuals spanning many generations. A pedigree analysis of a subset of deceased individuals represented in the San Diego Zoo Wildlife Alliance Biodiversity Bank’s Frozen Zoo^®^ revealed an underrepresented male that lived from 1975 to 1998, Studbook Number 615 (also known as Kuporovitch), who would be of high value for breeding if cloned. Here, we report that two healthy clones were produced from this cell line using cross-species somatic cell nuclear transfer from 2020 to 2023. Their identification as clones was verified by a standard horse-pedigree genotyping panel, and, for one clone, a whole genome sequencing comparison to the original donor was performed. This is the first time that multiple healthy clones surviving the perinatal period have been produced for an endangered species.

## Simple Summary

The Przewalski’s horse, *Equus przewalskii*, was saved from extinction by ex situ breeding and reintroduction into the wild. While the program has been very successful, the world’s ~3000 living Przewalski’s horses are descended from just 12 survivors captured from the wild between 1898 and 1947. Biobanking and assisted reproductive technologies offer means to improve the genetic management of the species and ensure viability throughout recovery. Towards that goal, we report the births of the first two clones produced from cells cryopreserved in 1980 using domestic horse oocytes and surrogate mothers via cross-species somatic cell nuclear transfer. The two male horses are clones of International Studbook Number (SB) 615, a stallion that lived from 1975 to 1998 that was underutilized in genetic management. The clones of SB615 present an opportunity to restore valuable founder genetics to the global population. This milestone represents the fifth endangered species ever successfully cloned and one of the first true applied conservation cloning efforts.

## 1. Introduction

Przewalksi’s horse, *Equus przewalskii*, was extirpated from its wild range across the central Eurasian grasslands with the last commonly cited wild sighting in the Dzungarian Gobi region in 1969 [1], at which time, according to the Przewalski’s Horse International Studbook, 174 individuals survived distributed across several European and North American zoos. Those animals were descended from several founder lines that had been maintained for display. Fortunately, by the 1950s, zoo managers recognized that the future of the species relied on the horses surviving in ex situ herds. In 1959, the first International Symposium on the Preservation of the Przewalski’s Horse was organized by the Prague Zoo, initiating the first efforts to establish a plan to not only save the species from extinction but to organize a coordinated breeding program to produce enough horses to begin restoring herds to the wild. The plan was formalized at the Fifth International Symposium in 1990 [2]. The breeding program had been rather successful and, following the Fifth Symposium, reintroductions began in the early 1990s. Today, several hundred horses live in free-roaming and semi-free-roaming herds in Mongolia, China, Russia, Kazakhstan, and Hungary. The world’s ~1800 living Przewalski’s horses are the result of over 8000 horses bred over nearly 20 generations from just 12 wild founders captured during 1898–1947 [3].

While the history of Przewalski’s horse is similar to many other species saved from extinction through conservation breeding and reintroductions, such as the California condor, whooping crane, or black-footed ferret, its breeding management history is complex due to its origins preceding the modern era of zoo conservation [4]. To illuminate by contrast, the founders of the endangered black-footed ferret breeding program were captured over a short period of time, brought into captivity together, and bred in a manner to attempt to retain as much genetic diversity as possible. The result is that all living black-footed ferrets today, save for three clones and their offspring [5], trace their ancestry to all founders in roughly equal proportions [6]. The Przewalski’s horse founders were captured over a long period of time, beginning in the 1890s, and distributed across several zoos in which they were bred as separate lineages for many decades before the coordinated efforts for conservation were initiated. The founders were not captured and bred with the intent to save the species nor necessarily to maximize the retention of surviving genetic diversity. Thus, when coordinated efforts for restoration began, the population was represented by five distinct lineages, two of which whose founders were interbred with domestic horses (*Equus caballus*) [7,8].

Even when concerted restoration breeding efforts began, there were significant differences in opinion as to how best to interbreed the five founder lineages. A desire for “genetic purity” led to continued and problematic segregation. Combined with breeding policies aimed at purging domestic horse ancestry within introgressed lines, these management practices led to significant losses of wild Przewalski’s horse genetic diversity and high inbreeding coefficients [9]. Experts at the time disagreed about the phenotypic impacts of the introgression of the Mongolian domestic mare, but to our knowledge, a comprehensive morphological analysis has never been reported. Fortunately, amidst this complex breeding history, initially uninformed by currently accepted practices of conservation management, tissue biopsies of a large number of historic Przewalski’s horses were collected and cryopreserved at multiple zoos, establishing a biobank today of nearly 600 individuals at the San Diego Zoo Wildlife Alliance Biodiversity Bank’s Frozen Zoo^®^ alone (298 individuals represented with cell lines and 277 represented by tissues preserved in DMSO from which cell lines could potentially be derived post-thaw, including reproductive tissues from 19 of the individuals mentioned above), with several individuals just 1–5 generations separated from the wild founders.

Many individuals with domestic horse ancestry were intentionally and detrimentally underutilized in the program, including Studbook Number (SB) 615, the donor sire of the clones reported here. A 1989 pedigree analysis concluded that significant historic allelic diversity would be lost without increasing representation of this lineage [7], which led to the decision to make breeding exceptions for SB615. However, despite those findings, SB615 was only bred during 4 out of 21 potential breeding seasons in his lifetime. From 1978 (the year SB615 reached sexual maturity and could have been bred) to 2023, a total of 8316 Przewalski’s horses were born in the conservation breeding program, of which only 61 (0.7%) descended from SB615. It was not until 2004 that the policy to purge domestic horse ancestry was completely abolished in favor of maintaining the maximum genetic diversity of the 12 wild Przewalski’s horse founders. Cloning offers a means to rescue some of the founder diversity that has been lost due to this history of problematic selective breeding.

**Figure 1:**
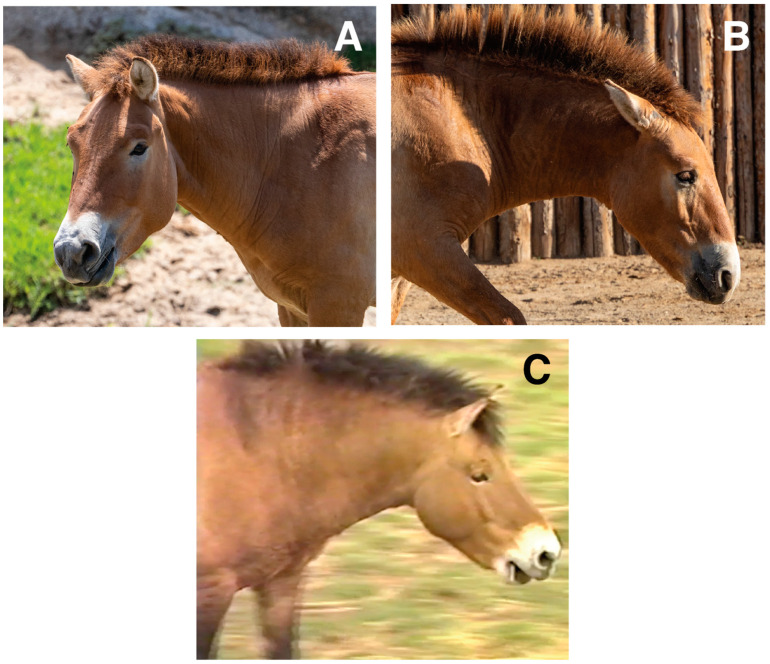
(**A**) Kurt (SB8268), the first clone born 6 August 2020, at 25 months of age. (**B**) Ollie (SB9029), the second clone, born 17 February 2023, at 7 months of age. Photographs A and B are cropped from originals provided in press release packages by San Diego Zoo Wildlife Alliance. (**C**) Still-frame from archival footage of SB615, Kuporovitch, 8 years of age.

We report the first clones of the endangered Przewalski’s horse, or Takhi, as it is called by Mongolians. The clones were produced via cross-species somatic cell nuclear transfer from donor cells cryopreserved in 1980 and using domestic horses (*Equus caballus*) as oocyte donors and surrogate mothers. The two males produced so far, named Kurt, International Studbook Number 8268 (SB8268) and Ollie, SB9029 (Figure 1A,B), are clones of SB615, Kuporovitch (Figure 1C), born in 1975 and died in 1998. Concerns regarding low genetic diversity, given the complex history of the species, prompted the effort to clone this historic and underrepresented stallion.

## 2. Methods

### 2.1. Pedigree Analysis

For the 2020 cloning attempt, biobanked cell lines were analyzed to determine which would confer the greatest genetic benefit to the cooperatively managed Association of Zoos and Aquariums’ (AZA) Przewalski’s horse population. Cooperative management for this population occurs through a strategy of minimizing pedigree-based kinships [10,11]. The mean kinship of an individual is the average kinship between that individual and all other living individuals in the population including itself. Reproduction from breeding pairs with low mean kinships and inbreeding coefficients below the population’s average mean kinship are prioritized. Twelve cell lines housed at the San Diego Zoo Wildlife Alliance Biodiversity Bank’s Frozen Zoo^®^ were each independently analyzed to compare their mean kinships, should they be added to the population, with the average mean kinship of the managed population living at the time of analyses. The population included both AZA and non-AZA facilities that were participating in cooperative management. Data from the AZA Regional Asian Wild Horse (*Equus przewalskii*) Studbook (current to 31 December 2019 [12]) was exported from Poplink 2.5.2 [13] to PMx v.1.6.20190628 [14] to calculate pedigree-based kinships. Sterilized males and females over the age of 25 were excluded from kinship analyses. Each cell line, independently of the others, was added to the genetically managed AZA population to estimate the cell line’s mean kinship within the population.

The kinship analysis to prioritize cell lines for the 2023 cloning attempt were performed using the same methods as in 2020 with the following modifications. Thirteen additional cell lines were added to the genetic analyses for a total of 25 cell lines examined. Data from the International Studbook for Przewalski’s horse was exported from the ZIMS Studbook (current to December 2021 [3]) to PMx v.1.7.0.20210915 to calculate pedigree-based kinships. In addition to comparing the mean kinship of the cell lines in the context of the AZA population that included the young 2020 clone, mean kinships of the cell lines within three other regional populations were examined: the European Association of Zoos and Aquaria (EAZA) population, the Eurasian Regional Association of Zoos and Aquariums (EARAZA) population, and the combined reintroduced populations of Orenburg Nature Reserve (Orenburg, Russia), Great Gobi B Strictly Protected Area (Altai-Khovd, Mongolia), Hortobagy National Park (Hortobagy, Hungary), and Hustai Nuruu National Park (Mongolia). To account for the possible genetic representation from future reproduction of the 2020 clone within the AZA population, 8 hypothetical offspring were added to the AZA population where the 2020 clone was the sire and 7 reproductive females residing at San Diego Zoo Safari Park were the dams.

### 2.2. Somatic Cell Nuclear Transfer

To clone SB615, a vial of the historically cryopreserved fibroblast cells was shipped from the San Diego Zoo Wildlife Alliance Biodiversity Bank’s Frozen Zoo^®^ (Escondido, CA, USA) to the commercial cloning company ViaGen Pets & Equine’s Texas-based facility (Cedar Park, TX, USA) where the cells were thawed and cultured at 37 °C in 5% CO_2_ in DMEM supplemented with 10% FCS with penicillin-streptomycin. Donor cells were fused with enucleated domestic horse oocytes to produce a total of 11 reconstructed embryos using the traditional method of injecting donor cells into the ooplasm. Oocytes were obtained by ovum-pickup. The reconstructed embryos were directly transferred to 11 domestic horse surrogate mares (1 embryo per mare). Young, large, reproductively sound and behaviorally gentle mares were selected as surrogates to ensure the safety of handlers and increase the likelihood of successful outcomes. Surrogates were monitored by ultrasound.

### 2.3. Genetic and Genomic Analyses

The identity of the clones was verified by a microsatellite panel of both clones and whole genome sequencing of one clone, Kurt (SB8268). Given that the clones were produced via cross-species somatic cell nuclear transfer, it was expected that their mitochondrial haplotypes should be a mismatch to the donor, which was verified by whole mitochondrial genome sequence assembled from the shotgun genome data of Kurt.

#### 2.3.1. Microsatellite Genotyping

FTA cards with 12–15 drops of whole blood from each clone were shipped to the University of California Davis Veterinary Medicine Veterinary Genetics Laboratory for nuclear microsatellite genotyping along with a cell pellet sample of the SB615 donor cells. A panel of 15 nuclear microsatellite loci were analyzed for each clone, which comprised the mandatory loci for assessing equine parentage recommended by the International Society for Animal Genetics (https://www.isag.us/, accessed on 1 February 2025) and other parentage markers: *AHT4*, *AHT5*, *AME*, *ASB2*, *ASB17*, *ASB23*, *HMS2*, *HMS3*, *HMS6*, *HMS7*, *HTG10*, *HTG4*, *LEX3*, *LEX33*, and *VHL20* [15].

#### 2.3.2. Whole Genome Analysis

Given the close evolutionary relationship and introgression between domestic and Przewalski’s horses, previous mtDNA studies have found shared haplotypes between the species [16,17], and, therefore, PCR and sequencing of a single mtDNA locus may not provide adequate resolution for identity comparisons. These factors may also obscure microsatellite analyses. Therefore, whole genome sequencing was performed to compare nuclear/mitochondrial DNA identities of one of the two clones, Kurt (SB8268), to the donor cells alongside a selection of additional unrelated Przewalski’s horses as a control data set. Cell pellets from cultured fibroblasts of each horse were shipped to the commercial service lab Psomagen, Inc. (Rockville, MD, USA) for short-read resequencing. Specifically, genomic DNA was extracted from cell pellets using the Mag-Bind Blood and Tissue Kit (Omega Bio-Tek Inc.; Norcross, GA, USA). DNA concentrations were assessed using Picogreen and Victor x2 fluorometry (Life Technologies, Carlsbad, CA, USA) and fragment quality was checked with an Agilent 4200 Tapestation (Agilent Technologies, Santa Clara, CA, USA) and a 1% TBE gel electrophoresis. Genomic DNAs were then sheared into 350 bp fragments with a Covaris S220 Ultrasonicator (Covaris, Woburn, MA, USA). DNA fragments were used to generate genomic libraries using the TruSeq DNA PCR-free library kit (Illumina, San Diego, CA, USA), which were quality checked on an Agilent 4200 Tapestation and quantitated using a Lightcycler qPCR assay (Roche Life Science, St. Louis, MO, USA). Libraries were pair-end sequenced (2 × 150 bp) to a target depth of 20X on an Illumina NovaSeq 6000 instrument (Illumina) with S4 flow cells, which generated ~56 Gb for each sample (Table 1).

Paired-end reads were explored with FastQC to calculate and visualize sequence quality metrics [18]. The reads were aligned to the reference genome assembly of domestic horse (https://www.ncbi.nlm.nih.gov/datasets/genome/GCF_002863925.1/, accessed on 1 February 2025) using the BWA-MEM- (v0.7.17) algorithm with default parameters [19], and the reference was indexed and a dictionary was created using SAMtools (v.1.17) [20] and Picard (v2.27.5) [21]. The mapped reads were sorted with SAMtools (v1.17), and duplicated reads were marked with Picard (v2.27.5) using MarkDuplicates. Finally, alignment statistics were evaluated with Picard and SAMtools. The median coverage was 18X, 18X, 17X, 19X, and 17X for Kurt, Kuporovich (SB615), Rosa (SB162), Bars (SB285), and Vintel (B1376), respectively (Table 1).

Single nucleotide variants (SNVs) were called using GATK HaplotypeCaller (v4.2.6.1) through a joint calling approach [22], followed by the use of GATK SelectVariants to obtain only autosomal SNVs. VariantFiltration was applied to filter the vcf files based on the following parameters: DP < 5.0”, “QD < 2.0”, “QUAL < 30.0”, “SOR > 3.0”, “FS > 60.0”, “MQ < 40.0”, “MQRankSum < −12.5“, “ReadPosRankSum < −8.0”.

The variant call set was further filtered by excluding multiallelic variants and private alleles analysis using BCFtools (v1.17) [20].

**Figure 2:**
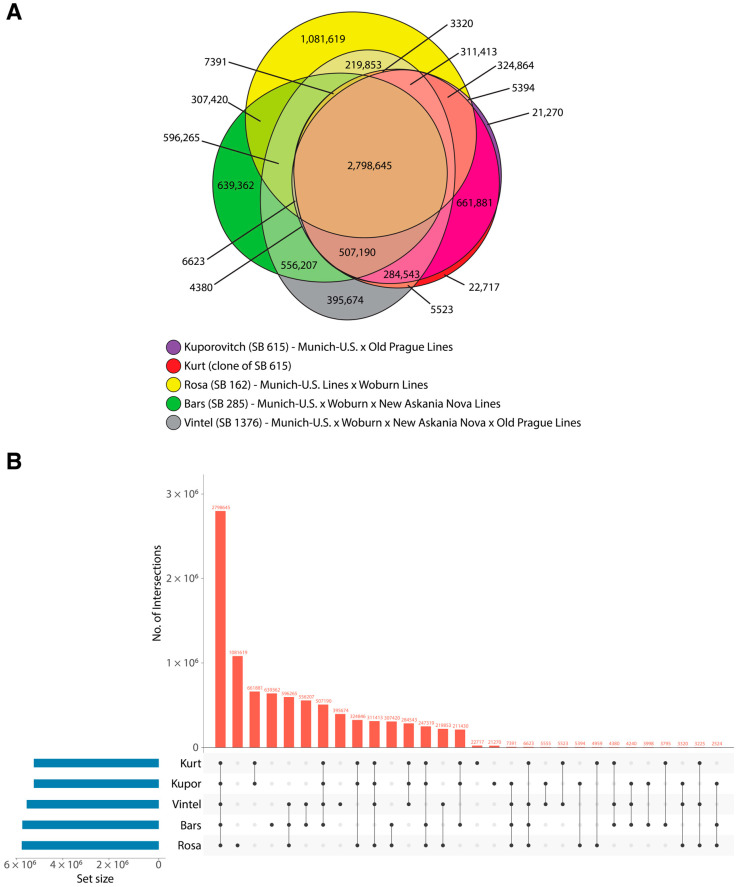
(**A**) Euler diagram and (**B**) upset plot illustrating biallelic private and shared single nucleotide variants (SNVs) for five Przewalski’s horses of multiple founder breeding lines, revealing a nearly identical overlap of Kurt (clone SB8268) and Kuporovitch (donor SB615).

To illustrate the proportions of private SNVs and those that are shared between individuals, a Euler diagram was created based on biallelic SNVs alone using the Eulerr R package [23] (Figure 2). In addition, an upset plot was generated using the Intervene (v0.6.5) tool [24] to examine the intersection of SNVs among the five individuals.

#### 2.3.3. Mitochondrial Genome Assembly and Analysis

The mitochondrial genomes of the five sequenced Przewalski’s horses were de novo assembled using GetOrganelle version 1.7.7.0 [25]. The full read set for each individual was used as input and the complete mitochondrial genome of the domestic horse was employed as the seed, with the -F animal_mt parameter and all remaining parameters set to their default values. The mean coverage of the assemblies ranged from 342 to 1619X, as estimated with Mosdepth [26]. We obtained mitochondrial genome assemblies ranging in length from 16,576 to 16,593 bp, with the length differences corresponding to the number of tandem repeats in the control region. The mitogenomes were annotated in the MITOS2 webserver [27].

To assess the phylogenetic position of the five Przewalski’s horses with respect to their maternal ancestry, particularly the cloned individual Kurt (SB8268), we downloaded mitochondrial genomes of available Przewalski’s horses (*n* = 16) as well as those from a diversity of domestic horse breeds (*n* = 32) from NCBI’s Genbank, which were published in previous studies [28,29,30,31]. We used the mitochondrial genome phylogeny reported by Der Sarkissian et al. [31] to select domestic horse breeds that provided the maximal representation of mitochondrial lineage diversity. We also downloaded the mitogenome of an onager, *Equus hemionus* (HM11885) [32], which was used as the outgroup to root the phylogenetic tree. All mitogenomes were imported into Geneious Prime version 2023.2 (https://www.geneious.com; accessed on 1 February 2025), after which a 16,748 bp multiple sequence alignment was generated using the MAFFT version 7.490 plugin with the following settings: AUTO algorithm (to select the optimal alignment strategy based on data set size), 200PAM/k = 2 scoring matrix, 1.53 gap open penalty, and an offset value of 0.123 [33]. Due to poor alignment and missing sequence data for some individuals in the control region, we trimmed 618 bp from this region, resulting in a final alignment of 16,130 bp. This alignment was used to construct a maximum likelihood phylogeny using the Geneious Prime plugin of RAxML version 8.2.11 [34]. The nucleotide substitution model was set as GTRGAMMA and the search algorithm was set as “Rapid bootstrapping and search for beast-scoring ML tree”, with the number of bootstrap replicates = 500, parsimony random seed = 3, and starting tree = random. The resulting tree was visualized in Geneious Prime with subsequent modifications performed in Microsoft PowerPoint.

## 3. Results

### 3.1. Pedigree Analysis Results

The results of the pedigree analysis are given in Table 2. SB615 (Cell Line #615 in the Table) was determined to be the most genetically valuable individual for cloning in 2020 based upon the ranking of mean kinship and sex. The studbook number identity of other cell lines is not disclosed as they are proprietary to multiple institutions. SB615 ranked 2nd only to CL#4, a female who happened to be the sister of SB615. Females were included in the pedigree analysis, but ideally males are more valuable for breeding given they can be paired with multiple females each breeding season and produce far more offspring during their lifetime than females. Given that SB615 should share significant genetic variation with CL#4 and he was a male, that line was selected for cloning.

For the 2023 analysis, which included more individuals and populations, SB615 ranked 4th, 3rd, 1st, and 11th overall for the AZA, EAZA, EARAZA, and reintroduced populations, respectively. SB615 was the highest ranking male for the AZA and EARAZA populations, while CL#23 ranked higher among the EAZA. The reintroduced population is the outlier in the analysis. Ten individuals outranked SB615: Cell Line #’s 1, 6, 7, 10, 13, 14, 16, 18, 21, and 22, of which seven were males. All of these cell lines ranked in the midrange, consistently below SB615 and his sister CL#4, as well as female Cell Line #’s 3 and 9. Overall, SB615 was consistently a valuable male again in 2023, even when considering 8 theoretical offspring of the 2020 clone for the AZA population. Therefore, the line was selected again for the second cloning attempt. Although considered minor in likelihood, the potential risk of injury, illness, or premature death of the 2020 clone was also a factor in the decision to produce a second clone from the SB615 cell line. Having two clones further mitigates potential risks to future reproduction and will increase the reproductive capacity of the lineage.

### 3.2. Somatic Cell Nuclear Transfer Results

A total of 7 of 11 recipient mares became pregnant, of which 2 resulted in live births (Table 3, Figure 1). The births proceeded naturally and required no interventions. The foals were born healthy; no veterinary interventions were necessary during pari-natal and post-natal development. The other five pregnancies were lost at various stages. The anatomy of all pre-term fetuses was analyzed and was developmentally normal. The loss of one pregnancy was determined to be premature placental separation, a condition which occurs in naturally conceived pregnancies. The causes of the other losses were indeterminate.

### 3.3. Whole Genome and Mitochondrial Genome Analyses Results

Overall, all genetic and genomic analyses support that the clones are a match to the donor cell line. The two clones were a complete match for the microsatellite panel. Regarding whole genome analysis, both the euler diagram and upset plots (Figure 2) showed a significant overlap between the clone (Kurt SB8268) and the donor (Kuporovitch, SB615) when compared to the other three individuals. A total of 9,250,892 SNVs were called across all five individuals, of which 5,454,492 SNVs were called for Kurt and Kuporovitch collectively; of those, 5,347,267 (98%) were identical. When compared to the other individuals, Kurt and Kuporovich possessed 22,717 and 21,270 private alleles, respectively; from a genetic point of view, the two individuals are equal [35,36]. The number of differences observed falls within the expected range when considering (1) mutations induced during cell culture of donor cells (the cells were cultured prior to SCNT as well as cultured to expand for genome sequencing), (2) somatic mutations in the developing clone, and (3) potential sequencing error. The number of private alleles shared across Kurt and Kuporovitch that are unique compared to the other three individuals is 661,881, comparable to the numbers of private alleles in other individuals which vary from 395,674 in Vintel SB1376 to 1,081,619 in Rosa SB162, representing the latest and earliest generations of the pedigree in our sample set, respectively.

**Figure 3:**
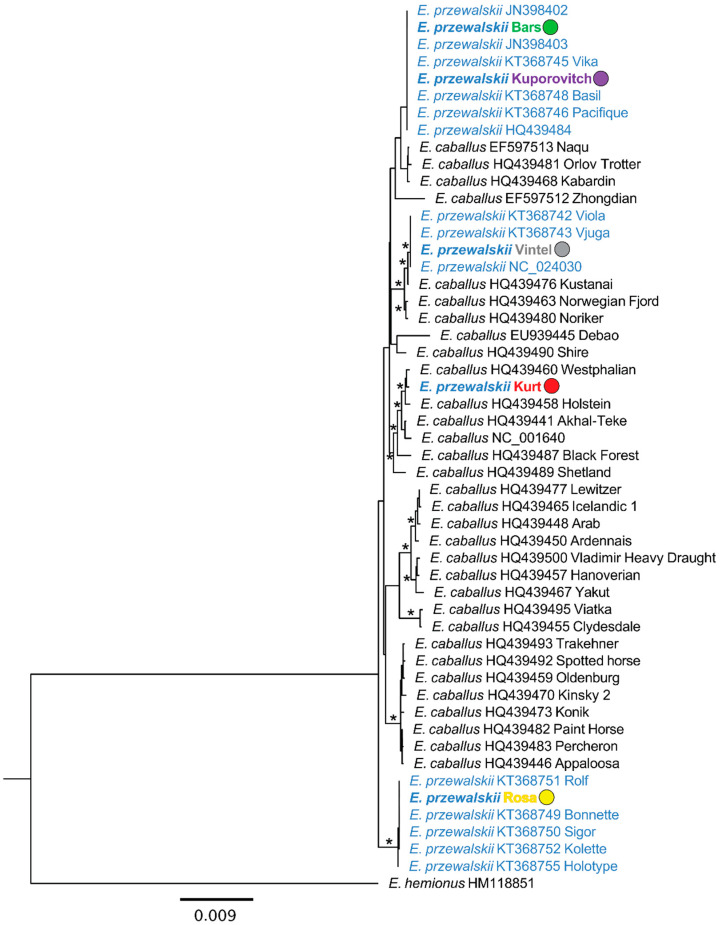
Maximum likelihood phylogenetic tree of mitochondrial genomes of domestic horses (black colored labels) and Przewalski’s horses (blue colored labels) based on an alignment of 16,130 bp. The tree is rooted with the onager (*Equus hemionus*). NCBI Genbank accession numbers are associated with domestic and Przewalski’s horses sequenced in previous studies. Asterisks indicate that out of 500 replicates, nodes with >90 bootstrap support the values. Przewalski’s horse individuals that were newly sequenced for this study are indicated by colored circles corresponding to Figure 2A, revealing the expected mismatch between Kurt (SB8268) and Kuporovitch (SB615). The scale bar at the bottom refers to the number of substitutions per site along branches.

Phylogenetic analysis of mitochondrial genomes confirmed that Kurt’s maternal ancestry is derived from a domestic horse, as his mitogenome is nested within a clade of domestic horses representing Westphalian, Holstein, Akhal-Teke, Black Forest, and Shetland breeds, with >90% bootstrap support across most nodes within the clade (Figure 3). In contrast, the four other individuals with an expected Przewalski’s horse maternal ancestry, Bars, Kuporovitch, Rosa, and Vintel, are each grouped within one of the three clades containing other Przewalski’s horse mitogenomes, which are supported by >90% bootstrap values. We note that the mitogenomes of Bars and Kuporovitch have identical haplotypes with the mitogenomes previously sequenced from these two individuals, KT368744 and KT368747, respectively.

## 4. Discussion

While several dozen domestic and wild species have been successfully cloned [37], according to the International Union for the Conservation of Nature’s (IUCN) assessment and classifications of endangerment (Near Threatened, Vulnerable, Endangered, Critically Endangered, Extinct in the Wild), the Przewalski’s horse was only the fifth endangered species to be successfully cloned and the first time that multiple viable clones of an endangered species survived past the neonatal period (Table 4; note several amphibian species [38,39,40,41,42] and one mammal, the cynomolgus monkey, *Macaca fascicularis* [43], that are currently assessed as endangered were not considered endangered at the time they were first cloned; and two mammals, the sand cat, *Felis margarita* [44], and the European mouflon, *Ovis aries musimon* [45], were erroneously considered endangered at the time of cloning but have since been reclassified). Since the birth of the Przewalski’s horse clones, there have been three black-footed ferret, *Mustela nigripes*, clones that have reached adulthood, making it the most abundantly cloned endangered species to date [46].

Recruitment of the SB615 cell line was deemed genetically beneficial, as analyses suggested that the cell line be underrepresented within multiple regionally managed conservation breeding programs (Table 2). This is only the second of three species in which decades-old biobanked materials have been used to produce clones for conservation management; the first being a Banteng, *Bos javanicus*, cloned from 25-year-old cells [47,49], the third being black-footed ferrets, *Mustela nigripes*, cloned from 32 year old cells. The clones represent the second opportunity in history for clones of an endangered species to fulfill their purpose: to reproduce and rescue valuable genetic diversity for conservation breeding [53,54,55]. A black-footed ferret clone recently achieved this milestone for the first time [5]. While neither of the Przewalski’s horse clones have yet reached sexual maturity during a breeding season event (Przewalski’s horse males reach sexual maturity at 4–5 years of age [56]), the testicular development of each clone is so far normal (personal communication, Hendrik Nolans, San Diego Zoo Wildlife Alliance).

The reproducible success of cloning this decades-long cryopreserved cell line demonstrates the potential to clone other cryopreserved cell lines for ongoing genetic management. Thanks to the extensive biobanking efforts of cell lines and germplasm (gametes) at zoos like San Diego Zoo Wildlife Alliance and the Smithsonian National Zoo & Conservation Biology Institute, cloning more historic individuals or “resurrecting” stallions through artificial insemination [57] offers the chance to ameliorate some of the genetic erosion caused by decades of managing the small zoo populations. In practice, the extensive biobank of Przewalski’s horse cell lines and gametes can now be viewed as an active reproductive resource for future breeding decisions. The use of advanced reproductive technologies is still rare in conservation, owing partially to the nascency of reproductive knowledge and techniques for most species [58]. While biobanking of viable cells and gametes for endangered species has been established in multiple centers, its potential impacts are, at this point limited, by the number of taxa cryopreserved and the small extent to which these collections capture extant/historic variation [59], despite well-established methods for many species, particularly for mammals [60]. The Przewalski’s horse recovery program could be the first to demonstrate the ideal model system for biobanking for conservation impact [54].

## 5. Conclusions

The birth and subsequent weaning of a second Przewalski’s horse clone marked the first time multiple viable clones were produced for an internationally listed endangered species. Although the total number of clones produced is low (*n* = 2), both attempts at cloning yielded success, demonstrating the reproducibility and reliability of cloning for genetic rescue purposes for the endangered Przewalski’s horse. This genetic rescue opportunity was only possible thanks to decades of biobanking efforts. Wide sampling and cryopreservation of cells and tissues across generations and lineages of the breeding program was key to capturing biomaterials that would one day have strategic genetic value, such as the cells of Kuporovitch (SB615) who lived from 1975 to 1998. When the clones become sexually mature between late 2025 and 2028, respectively, they will establish more breeding opportunities for an underrepresented lineage possessing disproportionate ancestry to 2 of the 12 founders, ensuring their contribution persists in future generations. Beyond Przewalski’s horses, the cross-species methodology used here establishes an effective means to apply cloning to the conservation of other endangered equids. More broadly, this work emphasizes the value of biobanking and reproductive sciences for aiding endangered species.

## Figures and Tables

**Table 1:** Raw data statistics.

Name/Studbook ID	Sample ID	Total Read Bases (bp)	Total Reads	GC (%)	Q20 (%)	Q30 (%)	Median Coverage *
Kurt SB8268	Kurt_clone	62,358,736,666	412,971,766	43.54	96.54	91.21	18X
Kuporovitch SB615	Kupor_KB4064	64,695,615,082	428,447,782	43.90	96.47	91.09	18X
Rosa SB162	Rosa_KB3838	65,253,774,804	432,144,204	43.49	96.22	90.48	19X
Bars SB285	Bars_KB7674	58,172,965,024	385,251,424	43.43	96.25	90.55	17X
Vintel SB1376	Vintel_KB9319	62,340,946,752	412,853,952	43.99	96.31	93.17	17X

* median coverage after mapping and removing duplicates.

**Table 2:** The mean kinship of each analyzed cell line (CL) relative to each population of N size, for which *n* individuals collectively have an average mean kinship (MK) listed. Cell line 615, chosen for both the 2020 and 2023 clones, is in bold. * Mean kinship of cell line 615 after siring eight hypothetical offspring. The identity of other cell line studbook numbers is not disclosed as they are proprietary to multiple institutions.

Cell Line ID	Cell Line Sex	N. America 2020 *n* = 121 MK = 0.2175	AZA 2023 *n* = 74 MK = 0.2046	EAZA *n* = 211 MK = 0.1659	EARAZA *n* = 106 MK = 0.1946	Reintroduced *n* = 1161 MK = 0.1658
**CL#615**	**M**	**0.0930**	**0.1137/0.1407** *	**0.1309**	**0.0913**	**0.1432**
CL#1	M	-	0.1848	0.1555	0.1563	0.136
CL#2	F	0.2322	0.2192	0.192	0.2047	0.1644
CL#3	F	-	0.1027	0.1346	0.0925	0.1466
CL#4	F	0.0842	0.0993	0.135	0.0927	0.1475
CL#5	M	0.1338	0.139	0.1561	0.1716	0.1589
CL#6	M	0.2211	0.2096	0.1567	0.1367	0.1316
CL#7	F	0.2091	0.1956	0.1581	0.1528	0.1308
CL#8	M	0.2437	0.2253	0.1788	0.1853	0.146
CL#9	F	-	0.097	0.1307	0.0928	0.146
CL#10	M	-	0.2243	0.1724	0.1777	0.1409
CL#11	M	0.2350	0.2184	0.1746	0.1801	0.1451
CL#12	M	-	0.196	0.1906	0.1899	0.1574
CL#13	M	0.2077	0.1949	0.1696	0.178	0.1395
CL#14	M	-	0.1962	0.1695	0.1852	0.1398
CL#15	F	-	0.2073	0.1847	0.1939	0.1531
CL#16	F	-	0.133	0.1382	0.1119	0.1382
CL#17	F	0.1335	0.138	0.1441	0.1127	0.1448
CL#18	F	0.2252	0.2111	0.1658	0.1613	0.1332
CL#19	M	-	0.1922	0.1838	0.186	0.1502
CL#20	M	-	0.1888	0.1817	0.1808	0.1509
CL#21	M	0.1945	0.1874	0.1562	0.1431	0.1404
CL#22	F	-	0.1253	0.1487	0.1176	0.1427
CL#23	M	-	0.1355	0.1241	0.1786	0.1578
CL#24	M	-	0.1287	0.1572	0.1475	0.1626
Difference between highest and lowest MK		0.1595	0.1283	0.0679	0.1134	0.0336

**Table 3:** Cross-species cloning results for donor cell line SB615. * Gestation includes 7 days in vitro.

Trial Year	Embryos Transferred	Surrogates	Pregnancies	Live Births (% Total Embryos)	Gestation Length *
2020	4	4	3 (75%)	1 (25%)	322 days
2023	7	7	4 (57%)	1 (14%)	337 days
Total	11	11	7 (63%)	2 (18%)	-

**Table 4:** History of cloning endangered species (limited to IUCN classifications). Number of live births and indivduals surviving the perinatal period are ordered according to the year.

Common Name	Species Name	IUCN Status	Year(s) Cloned	Number of Live Births	Number Surviving Perinatal Period	Reproductive Capability	References
Gaur	*Bos gaurus*	Vulnerable	2001, 2012	2	0	-	[47,48]
Banteng	*Bos javanicus*	Endangered	2003	2	1	Infertile (cryptorchidism)	[47,49]
Esfahan mouflon	*Ovis gmelini isphahanica*	Near Threatened	2010, 2015	3	Unknown	Not reported.	[50,51]
Argali	*Ovis ammon*	Near Threatened	2017	1	1	Fertile, produced hybrid offspring	[52]
Przewalksi’s horse	*Equus przewalskii*	Endangered	2020, 2023	2	2	Not yet sexually mature.	Reported here.
Black-footed ferret	*Mustela nigripes*	Endangered	2020, 2023	3	3	Fertile, produced offspring	[46]

## Data Availability

The genomic SRA datasets generated and/or analyzed during the current study are available in the National Center for Biotechnology Information repository under the BioProject PRJNA1098466 (https://www.ncbi.nlm.nih.gov/bioproject/PRJNA1098466, accessed on 1 February 2025).

## References

[1] Bouman D.T., Bouman J.G., Boyd L., Houpt D.A. (1994). The history of Przewalski’s Horse. Przewalski’s Horse: The History and Biology of an Endangered Species.

[2] Siefert S. (1992). Proceedings of the Fifth International Symposium on the Preservation of the Przewalski Horse.

[3] ZIMS for Studbooks for Przewalski’s Horse. (Simek, J. Prague Zoo, Current to January 2022). Species360 Zoological Information Management System. http://zims.Species360.org.

[4] Bolam F.C., Mair L., Angelico M., Brooks T.M., Burgman M., Hermes C., Hoffmann M., Martin R.W., McGowan P.J., Rodrigues A.S. (2021). How many bird and mammal extinctions has recent conservation action prevented?. Conserv. Lett..

[5] USFWS (2024). Advancements for Black-Footed Ferret Conservation Continue with New Offspring from Cloned Ferret. Press Release. https://www.fws.gov/press-release/2024-11/advancements-black-footed-ferret-conservation-continue-new-offspring-cloned.

[6] Marinari P., Lynch C. (2021). AZA Species Survival Plan® Yellow Program Population Analysis and Breeding and Transfer Plan for Black-footed Ferret (Mustela nigripes).

[7] Geyer C.J., Thompson E.A., Ryder O.A. (1989). Gene survival in the Asian wild horse (*Equus przewalskii*): II. Gene survival in the whole population, in subgroups, and through history. Zoo Biol..

[8] Bowling A.T., Zimmermann W., Ryder O., Penado C., Peto S., Chemnick L., Yasinetskaya N., Zharkikh T. (2004). Genetic variation in Przewalski’s horses, with special focus on the last wild caught mare, 231 Orlitza III. Cytogenet. Genome Res..

[9] Boyd L., Houpt K.A. (1994). Przewalski’s Horse: The History and Biology of an Endangered Species.

[10] Ballou J.D., Lacy R.C., Ballou J.D., Gilpin M., Foose T.J. (1995). Identifying Genetically Important Individuals for Management of Genetic Diversity in Pedigreed Populations. Population Management for Survival and Recovery.

[11] Fernandez J., Toro M.A. (1999). The use of mathematical programming to control inbreeding in selection schemes. J. Anim. Breed. Genet..

[12] Falino A. (2021). Asian Wild Horse (Equus przewalskii).

[13] Faust L.J., Bergstrom Y.M., Thompson S.D., Bier L. (2018). PopLink Version 2.5.2..

[14] Lacy R.C., Ballou J.D., Pollak J.P. (2012). PMx: Software package for demographic and genetic analysis and management of pedigreed populations. Methods Ecol. Evol..

[15] Bowling A.T., Eggleston-Stott M.L., Byrns G., Clark R.S., Dileanis S., Wictum E. (1997). Validation of microsatellite markers for routine horse parentage testing. Anim. Genet..

[16] Goto H., Ryder O.A., Fisher A.R., Schultz B., Kosakovsky Pond S.L., Nekrutenko A., Makova K.D. (2011). A massively parallel sequencing approach uncovers ancient origins and high genetic variability of endangered Przewalski′s horses. Genome Biol. Evol..

[17] Vilstrup J.T., Seguin-Orlando A., Stiller M., Ginolhac A., Raghavan M., Nielsen S.C.A., Weinstock J., Froese D., Vasiliev S.K., Ovodov N.D. (2013). Mitochondrial Phylogenomics of Modern and Ancient Equids. PLoS ONE.

[18] Andrews S., Krueger F., Segonds-Pichon A., Biggins L., Krueger C., Wingett S. (2010). FastQC. A Quality Control Tool for High Throughput Sequence Data. http://www.bioinformatics.babraham.ac.uk/projects/fastqc/.

[19] Li H., Durbin R. (2009). Fast and accurate short read alignment with Burrows–Wheeler transform. Bioinformatics.

[20] Danecek P., Bonfield J.K., Liddle J., Marshall J., Ohan V., Pollard M.O., Whitwham A., Keane T., McCarthy S.A., Davies R.M. (2021). Twelve years of SAMtools and BCFtools. GigaScience.

[21] Broad Institute (2021). Picard Tools. Broad Institute, GitHub Repository. http://broadinstitute.github.io/picard/.

[22] Poplin R., Ruano-Rubio V., DePristo M.A., Fennell T.J., Carneiro M.O., Van der Auwera G.A., Kling D.E., Gauthier L.D., Levy-Moonshine A., Roazen D. (2018). Scaling accurate genetic variant discovery to tens of thousands of samples. bioRxiv.

[23] Gibson T. (2021). Eulerr: Area-Proportional Euler and Venn Diagrams with Circles or Ellipses. R Package Version 6.1.1. https://cran.r-project.org/web/packages/eulerr/vignettes/introduction.html.

[24] Khan A., Mathelier A. (2017). Intervene: A tool for intersection and visualization of multiple gene or genomic region sets. BMC Bioinform..

[25] Jin J.J., Yu W.B., Yang J.B., Song Y., DePamphilis C.W., Yi T.S., Li D.Z. (2020). GetOrganelle: A fast and versatile toolkit for accurate de novo assembly of organelle genomes. Genome Biol..

[26] Pedersen B.S., Quinlan A.R. (2018). Mosdepth: Quick coverage calculation for genomes and exomes. Bioinformatics.

[27] Donath A., Jühling F., Al-Arab M., Bernhart S.H., Reinhardt F., Stadler P.F., Middendorf M., Bernt M. (2019). Improved annotation of protein-coding genes boundaries in metazoan mitochondrial genomes. Nucleic Acids Res..

[28] Xu S., Luosang J., Hua S., He J., Ciren A., Wang W., Tong X., Liang Y., Wang J., Zheng X. (2007). High altitude adaptation and phylogenetic analysis of Tibetan horse based on the mitochondrial genome. J. Genet. Genom..

[29] Lippold S., Matzke N.J., Reissmann M., Hofreiter M. (2011). Whole mitochondrial genome sequencing of domestic horses reveals incorporation of extensive wild horse diversity during domestication. BMC Evol. Biol..

[30] Achilli A., Olivieri A., Soares P., Lancioni H., Hooshiar Kashani B., Perego U.A., Nergadze S.G., Carossa V., Santagostino M., Capomaccio S. (2012). Mitochondrial genomes from modern horses reveal the major haplogroups that underwent domestication. Proc. Natl. Acad. Sci. USA.

[31] Der Sarkissian C., Ermini L., Schubert M., Yang M.A., Librado P., Fumagalli M., Jónsson H., Bar-Gal G.K., Albrechtsen A., Vieira F.G. (2015). Evolutionary genomics and conservation of the endangered Przewalski′s horse. Curr. Biol..

[32] Luo Y., Chen Y., Liu F., Jiang C., Gao Y. (2011). Mitochondrial genome sequence of the Tibetan wild ass (*Equus kiang*). Mitochondrial DNA.

[33] Katoh K., Standley D.M. (2013). MAFFT Multiple Sequence Alignment Software Version 7: Improvements in Performance and Usability. Mol. Biol. Evol..

[34] Stamatakis A. (2014). RAxML version 8: A tool for phylogenetic analysis and post-analysis of large phylogenies. Bioinformatics.

[35] Klinger B., Schnieke A. (2021). 25th anniversary of cloning by somatic-cell nuclear transfer Twenty-five years after Dolly: How far have we come?. Reproduction.

[36] Kim H.M., Cho Y.S., Kim H., Jho S., Son B., Choi J.Y., Jang G. (2013). Whole genome comparison of donor and cloned dogs. Sci. Rep..

[37] Jivanji S., Harland C., Cole S., Brophy B., Garrick D., Snell R., Laible G. (2021). The genomes of precision edited cloned calves show no evidence for off-target events or increased de novo mutagenesis. BMC Genom..

[38] Tung T.C., Wu S.C., Tung Y.Y.F., Yan S.Y., Tu M., Lu T.Y. (1963). Nuclear transplantation in fish. Sci. Bull. Acad. Sin..

[39] Sambuichi H. (1957). The roles of the nucleus and the cytoplasm in development I. An interspecific hybrid frog, developed from a combination of *Rana nigro-maculata nigromaculata* cytoplasm with a diploid nucleus of *Rana nigromaculata brevipoda*. J. Sci. Hiroshima Univ. Ser. B. Div..

[40] Signoret J. (1965). Transplantations nucléaires et différenciation embryonnaire. Arch. Biol..

[41] Aimar C. (1972). Analyse par la greffe nucléaire des propriétés morphogénétiques des noyaux embryonnaires chez Pleurodeles waltlii (*Amphibien urodèle*). Application à l’étude de la gémellité expérimentale. Ann. Embryol. Morphogen..

[42] Nishioka M. (1972). Reciprocal nucleo-cytoplasmic hybrids between *Rana brevipoda* and *Rana plancyi chosenica*. Sci. Rep. Lab. Amphib. Biol. Hiroshima Univ..

[43] Liu Z., Cai Y., Wang Y., Nie Y., Zhang C., Zu Y., Zhang X., Lu Y., Wang Z., Poo M. (2018). Cloning of macaque monkeys by somatic cell nuclear transfer. Cell.

[44] Gómez M.C., Pope C.E., Kutner R.H., Ricks D.M., Lyons L.A., Ruhe M., Dumas C., Lyons J., López M., Dresser B.L. (2008). Nuclear transfer of sand cat cells into enucleated domestic cat oocytes is affected by cryopreservation of donor cells. Cloning Stem Cells.

[45] Loi P., Ptak G., Barboni B., Fulka J., Cappai P., Clinton M. (2001). Genetic rescue of an endangered mammal by cross-species nuclear transfer using post-mortem somatic cells. Nat. Biotechnol..

[46] Novak B.J., Gober P., Bortner R., Garelle D., Wright M., Novak J., Houck M.L., Ryder O.A., Milutinovich D., Benavidez J. (2024). First endangered black-footed ferrets, *Mustela nigripes*, cloned for genetic rescue. bioRxiv.

[47] Friese C. (2013). Cloning Wildlife: Zoos, Captivity, and the Future of Endangered Animals.

[48] Srirattana K., Imsoonthornruksa S., Laowtammathron C., Sangmalee A., Tunwattana W., Thong Prapai T., Chaimongkol C., Ketudat-Cairns M., Parnpai R. (2012). Full-term development of gaur-bovine interspecies somatic cell nuclear transfer embryos: Effect of Trichostatin A treatment. Cell. Reprogramm..

[49] Janssen D.L., Edwards M.L., Koster J.A., Lanza R.P., Ryder O.A. (2004). 206 Postnatal management of cryptorchid banteng calves cloned by nuclear transfer utilizing frozen fibroblast cultures and enucleated cow ova. Reprod. Fertil. Dev..

[50] Hajian M., Hosseini S.M., Forouzanfar M., Abedi P., Ostadhosseini S., Hosseini L., Moulavi F., Gourabi H., Shahverdi A.H., Taghi Dizaj A.V. (2011). “Conservation cloning” of vulnerable Esfahan mouflon (*Ovis orientalis isphahanica*): In vitro and in vivo studies. Eur. J. Wildl. Res..

[51] Dehghan S.K. (2015). Scientists in Iran Clone Endangered Mouflon-Born to Domestic Sheep. The Guardian..

[52] Gomez J., Limehouse J. (2024). Montana Rancher Gets 6 Months in Prison for Creating Hybrid Sheep for Captive Hunting. USA TODAY..

[53] Ryder O.A., Benirschke K. (1997). The potential use of “cloning” in the conservation effort. Zoo Biol..

[54] Ryder O.A. (2002). Cloning Advances and Challenges for Conservation. Trends Biotechnol..

[55] Ballou J.D., Lacy R.C., Traylor-Holzer K., Bauman K., Ivy J.A., Asa C. (2023). Strategies for establishing and using genome resource banks to protect genetic diversity in conservation breeding programs. Zoo Biol..

[56] Monfort S.L., Arthur N.P., Wildt D.E., Boyd L., Houpt K.A. (1994). Reproduction in the Przewalski’s Horse. Przewalski′s Horse: The History and Biology of an Endangered Species.

[57] Pukazhenthi B.S., Johnson A., Guthrie H.D., Songsasen N., Padilla L.R., Wolfe B.A., da Silva M.C., Alvarenga M.A., Wildt D.E. (2014). Improved sperm cryosurvival in diluents containing amides versus glycerol in the Przewalski’s horse (*Equus ferus przewalskii*). Cryobiology.

[58] Comizzoli P., Holt W.V. (2019). Breakthroughs and new horizons in reproductive biology of rare and endangered animal species. Biol. Reprod..

[59] Mooney A., Ryder O.A., Houck M.L., Staerk J., Conde D.A., Buckley Y.M. (2023). Maximizing the potential for living cell banks to contribute to global conservation priorities. Zoo Biol..

[60] Houck M.L., Lear T.L., Charter S.J., Arsham M., Barch M., Lawce H. (2017). Animal Cytogenetics, Chapter 24. The AGT Cytogenetics Laboratory Manual.

